# Applying a microfluidic device to improve the Ca^2+^ separation performance of the liquid–liquid extraction process

**DOI:** 10.1038/s41598-022-26529-9

**Published:** 2022-12-20

**Authors:** Seyed Sajjad Jazayeri, Afham Pourahmad, Amin Hassanvand, Mozhgan Mozhdeh, Goodarz Tahmasbi

**Affiliations:** 1 Department of Chemical Engineering, Abadan Branch, Islamic Azad University, Abadan, Iran; 2 Department of Polymer Engineering, Amirkabir University of Technology (Tehran Polytechnic), Tehran, Iran; 3 Department of Polymer Engineering, Faculty of Engineering, Lorestan University, Khorramabad, Iran; 4 Petroleum and Chemical Engineering Faculty, Islamic Azad University, Science and Research Branch, Tehran, Iran; 5 Engineering Department, Azarbaijan Shahid Madani University, East Azarbaijan, Iran

**Keywords:** Other nanotechnology, Chemical engineering

## Abstract

This study investigates the application of extraction solvent in a new microfluidic apparatus to separate calcium ions (Ca^2+^). Indeed, a serpentine microfluidic device has been utilized to separate calcium ions. The flow regime map shows that it is possible to completely separate organic and aqueous phases using the serpentine microfluidic device. The suggested microfluidic device reaches the extraction efficiency of 24.59% at 4.2 s of the residence time. This research also employs the Box–Behnken design (BBD) strategy in the response surface methodology (RSM) for performing the modeling and optimization of the suggested extraction process using the recorded experimental data. Flow rate and pH of the aquatic phase as well as Dicyclohexano-18-crown-6 (DC18C6) concentration are those independent features engaged in the model derivation task. The optimum values of pH 6.34, the DC18C6 concentration of 0.015 M, and the flow rate = 20 µl/min have been achieved for the aquatic phase. The results indicated that the extraction efficiency of Ca^2+^ is 63.6%, and microfluidic extraction is 24.59% in this optimum condition. It is also observed that the microfluidic extraction percentage and experimental efficiency achieved by the suggested serpentine microchannel are higher than the previous separation ranges reported in the literature.

## Introduction

Calcium ions (Ca^2+^) and their enriched isotopes (^48^Ca, ^46^Ca, ^44^Ca, ^43^Ca, ^42^Ca, and ^40^Ca) are important components with a wide range of applications in different processes, including reducing agents for extracting metallic substances, allying agents for the metal production, and nutritional and medical studies^1–3^. Among various isotopes of calcium, ^48^Ca is extensively used in the absorption of calcium in the body and nutritional studies. In addition, other applications of this isotope contain tracking the migration of elements in plants and soil^4–6^.

The molecular laser processes, gaseous diffusion, gas centrifuges, electromagnetic, fractional distillation, and thermal diffusion are the most well-known scenarios often applied to separate stable isotopes^7–11^. Since electromagnetic and thermal diffusion are complicated and expensive scenarios to enrich ^48^Ca, the chemical exchange process appears as one of the important methods for separating the calcium isotope^12–16^.

Liquid–liquid extraction (LLE) is a suitable technique to improve the separation processes^17–21^. The organic solvent and the aquatic solution are often utilized as immiscible phases in this method. Because of toxic substances, flammability, and volatile organic compounds (VOCs), liquid–liquid extraction is the safe and reasonable method and it is also known as the environmentally friendly separation technique^22, 23^. Furthermore, process safety and solvent cost are the greatest complications that must be considered when a large-scale production is required^24–26^. Since the maximum value of the achievable separation parameter in the single-stage chemical exchange is ~ 1 × 10^–3^, it is necessary to increase the number of stages to reach a higher separation efficiency. This increase in the number of stages requires a bigger space and increases the capital cost and the operating time. In addition, the conventional methods of handling the extraction-based separation have several drawbacks, for instance, high response time and energy consumption level^27, 28^. In addition, this conventional method needs a mandatory extra stage to separate the phases (i.e., settling unit)^29^. To resolve these drawbacks, the microfluidic device can be used for accomplishing liquid–liquid extraction.

Microfluidic devices and microchannels have extensively been engaged in the LLE of a broad range of substances^30–33^. The most crucial advantage of these micro-scale devices compared with the conventional techniques is their high surface-to-volume ratio, which leads to reducing the diffusion length and increasing the mass transfer rate through the interface of phases^34–38^. Consequently, these micro-scale devices are suggested as an efficient technique for improving the separation performance of chemical and chemistry processes suffering from low throughput. In addition, these micro-size devices are highly safe, have a compact structure and well-defined hydrodynamic behavior, and possess complete µSX that further approve their potential application in the separation scenarios^39–42^.

Some researchers have investigated the application of the microfluidic device for separation purposes^43–51^. Helle et al. applied microfluidic devices to extract uranium (VI) with Aliquat^®^ 336 from HCl media^52^. After complete analyzing the effect of involved parameters, it was observed that both the batch and micro-size devices have almost the same performance. The authors have also conducted some mathematical analyses on coupling extraction and stripping micro-size units in a continuous mode.

Logtenberg et al. suggested a surface modification procedure to control two-phase flow in a polymeric micro-scale device^53^. This method not only adjusts both sides of the channel, but it also could easily control the behavior of two immiscible phases by the flow rate. The extraction of 2-butanol from the toluene/water/2-butanol system at different operating conditions has been investigated by Jovanovic et al.^54^. The experimental setup flows the water/toluene stream in extended capillary micro-scale reactors. The authors mainly focused on investigating the capillary length on the flow map in the micro-size reactor. The authors stated that the capillary length has a slight effect on the slug as well as bubbly flow regimes. Raimondi et al. introduced a mechanism to explain the mass transfer behavior of the liquid–liquid in the square microchannels with the slug flow regime^55^.

Marsousi et al. investigated the possibility of utilizing ionic liquids for enhancing the calcium extraction performance of spiral and Y-shape microfluid devices^23^. The results approved that the spiral micro-scale channel is a better candidate for calcium extraction when the ionic liquid and water flow rates are at their highest possible value. The same calcium recovery factor (i.e., 52%) has been achieved in the experimental and equilibrium conditions for these highest flow rates. An efficient technique for the separation of substances with a low separation tendency (like Ca^2+^ ion) has been introduced by Abdollahi et al.^56^. The Box–Behnken design (BBD) strategy of the response surface methodology (RSM) is applied to accomplish both the modeling and optimization tasks. The flow rate of the aquatic phase and its pH and DC18C6 (Dicyclohexano-18-crown-6) content are those independent features engaged in the modeling/optimization stage. The authors reported 5.1, 0.014 M, and 20 µl/min as the optimum values of the pH, DC18C6 concentration, and flow rate, respectively. The extraction efficiency of 62.28% has been achieved for the Ca^2+^ separation in this optimum condition. Heydarzadeh et al. investigate the salt-assisted LLE in a microchannel system^57^. They could increase the contact surface area between target analytes and the extracting phase during the sample and extracting phase transfer in the microchannel. Tang et al. experimentally studied the flow patterns and mass transfer characteristics of immiscible fluids based on droplet flow in a vertical microchannel^58^. An opposite-flowing T-shaped microchannel has been proposed to form favorable monodispersed droplet flow in a wide range of volume flow rate ratios. Singh et al. compare the performance of microchannels and conventional stage-wise extractors for LLE by using a standard phase system^59^. Three different microchannels—a T-junction microchannel, a serpentine microchannel, and a split-and-recombine microchannel—have been used in the experiments. Conventional extractors are represented by a mixer settler and an annular centrifugal extractor.

In two different studies, Jahromi et al. analyzed the LLE performance in Y-shaped micro-scale junctions^60, 61^. The first one introduced a mathematical approach that was derived via the interfacial pressure balance^60^. The results showed that the flow rate of the organic phase in the tube outlet should be adjusted in a value between the range suggested by the mathematical approach. Another research by Jahromi et al. proposed an efficient scenario for separating the calcium ions, i.e., continuous micro-solvent extraction reaction^61^. The RSM has also been applied to investigate the effect of main process parameters on the Ca^2+^ separation and find the optimized behavior of the process. The authors claimed that the reagent concentration is a significant factor that governs the separation efficiency. Indeed, increasing the calcium chloride concentration results in decreasing the separation efficiency. In addition, the existence of a small amount of DC18C6 and picric acid in the organic phase facilitates the extraction reaction. It has also been reported that the Ca^+2^ separation level intensifies by increasing contact time as well as flow rates.

This current research comprehensively investigates the application of a single-stage serpentine microfluidic device for Ca^2+^ separation via a micro-solvent extraction scenario. The key features of this separation scenario (i.e., microfluidic extraction percentage and extraction efficiency) at different operating conditions of a micro-size instrument are investigated. The effect of pH and flow rate of the aquatic phase and the DC18C6 concentration in the organic phase on the performance of the extraction process is also examined systematically. A great deal of effort is also made to optimize the process characteristics in such a way that it provides full-phase separation. The BBD mode of the RSM is applied to solve the optimization problem and find the optimum conditions. The results justified that the proposed serpentine micro-size channel has a higher microfluidic extraction percentage and experimental efficiency than those suggested in the literature.

## Materials and methods

### Materials

To achieve experimental investigations, picric acid, 98.0% pure DC18C6, sodium hydroxide (NaOH), and 99.9% pure CaCl_2_ (anhydrous Calcium chloride) beads with a mesh size of lower than 10 were purchased (Sigma Aldrich, Germany). The aquatic solution was made by mixing 0.01 M picric acid, deionized water, and 0.005 M CaCl_2_. On the other hand, the organic phase was prepared by mixing three different concentrations of DC18C6 (0.005, 0.01, and 0.015 M) with the organic solvent. Also, the aqueous phase was adjusted to the favorite values (8, 5.5, and 3) by adding NaOH to the initial pH. For the organic phase, we used an anhydrous *N*-butyl acetate with a purity of more than 99%, based on the previous experiment^56^.

The Ca^+2^ molar concentration in the aquatic phase is determined by analyzing 1 cc of the aqueous phase by Vista-Pro Inductively Coupled Plasma Optical Emission Spectroscopy (ICP-OES) apparatus.

### Equilibrium behavior of the LLE

The equilibrium behavior of the extraction samples is monitored in the volumetric flask. For 120 min, the same proportion of each phase was taken and magnetically mixed at an angular speed of 600 rounds per minute (rpm). This 120 min of mixing is done to ensure that the process experiences the equilibrium state. After that, two immiscible phases of a uniform, well-mixed dispersion were attained. Here, the convection mechanism dominates the mass transfer behavior of the organic/aquatic phase.

Utilizing the small angular speed for separating *n*-butyl acetate and water-dominated phases results in suspending small droplets of the organic phase in the aquatic phase which adversely affects the concentration measurement by the ICP apparatus. Therefore, this study employs a laboratory centrifugal device (for the mixture centrifugation with an angular speed of 3000 rpm for 5 min) to separate organic and aquatic phases. Afterward, the aquatic falcon phase was gathered by a syringe and caused to flow into sample vessels to measure the concentration.

### LLE in the micro-size device

**Figure 1:**
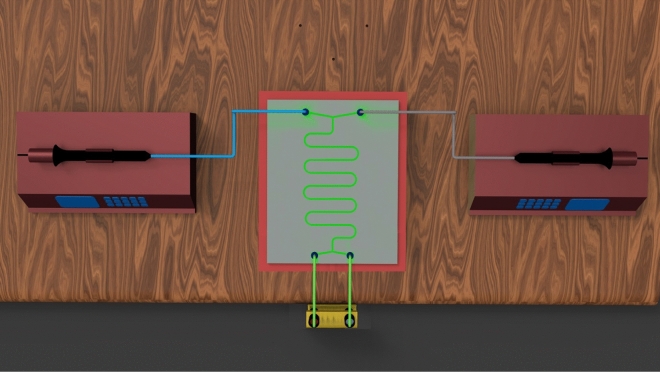
Schematic diagram of the laboratory-scale micro-size device.

Figure 1 presents our laboratory-scale microfluidic solvent extraction setup. This setup can be used to conduct the LLE experiments in a parallel flow mode in the microchannel serpentine apparatus (serpentine chip of 6 cm in length). The microfluidic chip was prepared with four gaskets and two grooved aluminum plates and connectors. Two high-precision programmable syringe pumps that inject organic and aquatic phases into the serpentine chip are responsible for the connection adjustment in a micro and macro interval. A connected Canon EOS 700D camera to a personal computer, light source, and microscope has been applied to simultaneously capture and save videos and images. Then, it is possible to continuously monitor the flow pattern at the outlet of the serpentine chip.

This research study uses the mechanical micromachining process to fabricate glass microchips through a computer numerical control device. The wide, height, and length of the microfluidic chip are 500 µm, 90 µm, and 6 cm, respectively.

### Modeling stage

#### Microfluidic extraction percentage

The separated value of Ca^+2^ in the microfluidic apparatus which is also known as the microfluidic extraction percentage and the yield of extraction is defined by Eq. (1).

1 $$\%E=100\times \frac{{[C]}_{aq,i}-{[C]}_{aq,o}}{{[C]}_{aq,i}},$$

where, the Ca^+2^ molar concentration in the inlet and the outlet aquatic streams of the serpentine microchannel abbreviated by the ${[C]}_{aq,i}$ and ${[C]}_{aq,o}$, respectively.

#### Extraction efficiency

The extraction efficiency is a significant parameter that exhibits the performance of a micro-size separation apparatus in comparison with the equilibrium condition. The mathematical form of the extraction efficiency is given by Eq. (2).

2 $${\%E}_{eff}=100\times \frac{{[C]}_{aq,o}-{[C]}_{aq,i}}{{[C]}_{aq}^{*}-{[C]}_{aq,i}},$$

where ${[C]}_{aq}^{*}$ indicates the Ca^+2^ molar concentration of the entry aquatic phase at the equilibrium condition.

### Optimizing the process

In the extraction efficiency, many parameters are impressive of Ca^+2^ with a micro-size separation apparatus. The RSM provides a user-friendly environment to monitor the effect of several variables of many responses (i.e., multi-input and multi-output problems). This technique includes a set of mathematical and statistical equations that help develop a reliable model from the experimental data. This technique is also able to reduce the number of experiments and solve the optimization problem of a multi-variable system^62–65^. This research uses a three-level BBD design of the RSM to determine the best conditions for doing experiments, derive models to relate responses to the independent features and find the optimum condition of the separation process. Equation (3) presents the general form of the second-order polynomial equation for correlating a response (Y) to independent variables (X_i_)^66–68^:

3 $$Y={\beta }_{0}+\sum_{i=1}^{k}{\beta }_{i}{X}_{i}+\sum_{i=1}^{k}{\beta }_{ii}{X}_{i}^{2}+\sum_{i=1}^{k-1}\sum_{j=2}^{k}{\beta }_{ij}{X}_{i}{X}_{j}+\varepsilon .$$

The adjustable coefficients of this quadratic model are ${\beta }_{0}$, ${\beta }_{i}$, ${\beta }_{ii}$, and ${\beta }_{ij}$. The $k$ and $\varepsilon$ symbols stand for the number of independent features and the model error, respectively. This study relies on the results of the analysis of variance (ANOVA) to appraise the accuracy of suggested regressive models. In addition, the P-value and F-test can be used to rank the importance of each independent feature or the combination of features on the considered response.

Here, the aquatic phase pH and flow rate (Q) and the DC18C6 concentration in the organic phase (C) were carefully chosen as independent variables for experimental and modeling investigating the separation performance of the microfluidic tool. The impact of these influential factors on the microfluidic extraction percentage $(\%E)$ and extraction efficiency $({\%E}_{eff})$ of the serpentine microfluidic system as the anticipated responses are assessed both experimentally and numerically.

Table 1 introduced the range of these independent variables as well as coded the variable levels. Table 2 summarizes the information of the 17 tests suggested by the DOE (design of experiment) to experimentally check in the micro-size extraction device. Moreover, five replicated tests were appended to the experiments to reduce the effect of random errors.

**Table 1:** The complete information on the design of the experiment.

Feature name	Indicator	Features’ level		
		High bound (+ 1)	Center (0)	Low bound (− 1)
Q (µl/min)	A	50	35	20
C (mol/l)	B	0.015	0.01	5 × 10^–3^
pH	C	8	5.5	3

**Table 2:** The obtained values for the %E and %E_eff_ via the micro-size device.

Run number	Factor A Q $(\mathrm{\mu l}/\mathrm{min})$	Factor B C $(\mathrm{mol}/\mathrm{l})$	Factor C pH (−)	First response E (%)	Second response E_eff_ (%)
1	35	0.01	5.5	13.92	37.46
2	35	0.005	3	6.16	23.05
3	35	0.01	5.5	15.23	38.19
4	50	0.01	8	13.69	34.91
5	35	0.01	5.5	15.38	40.02
6	35	0.01	5.5	15.51	39.98
7	50	0.015	5.5	13.85	30.09
8	35	0.005	8	14.16	37
9	20	0.01	3	13.97	55.31
10	20	0.01	8	20.43	52.61
11	35	0.015	3	6.64	32.68
12	35	0.01	5.5	14.92	40.13
13	20	0.005	5.5	16.85	44.76
14	50	0.005	5.5	12.32	32.84
15	50	0.01	3	2.81	7.19
16	35	0.015	8	15.43	39.24
17	20	0.015	5.5	24.59	63.6

## Results and discussions

### Selecting the working solvent

Similar to the previous research^56^, we also used *n*-butyl acetate as the working fluid in the current research. This selection helps us to compare the extraction performance of the proposed micro-size separation device with those reported in the literature.

### Flow regime map

**Figure 2:**
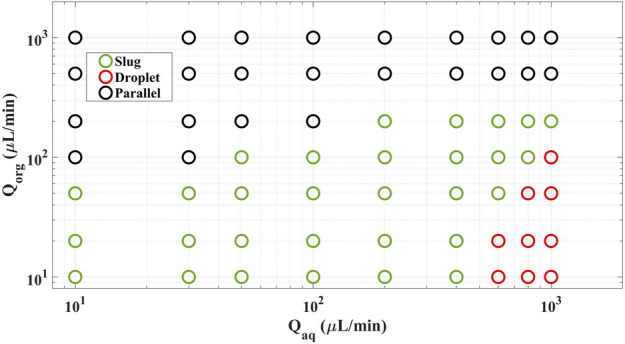
Flow regime map for the serpentine microfluidic (org and aq subscripts are aquatic and organic phases).

Two main flow patterns which often appeared in micro-size separation apparatus are slug and parallel regimes. Since the latter mode could handle a higher flow rate of the aquatic/organic phase, in this study we use the parallel flow regime to analyze mass transfer and hydrodynamics of the proposed microfluidic setup. In these situations, viscous forces dominate those related to the interfacial surface tension^43, 56^. Figure 2 shows the flow map of the serpentine microfluidic device. As can be seen from the figure, in the high flow rate of the organic and the aqueous phases, the flow map is parallel.

### RSM package

Although it is possible to apply the BBD strategy in many commercial packages, this research employs Design-Expert software (Version 13). This software efficiently derives the model, examines the experimental records, and finds optimum conditions of the considered process.

#### Model construction

This section uses the RSM to perform advanced regression analysis on the experimental data obtained from the serpentine microfluidic system. Indeed, numerous mathematical models were derived by adjusting their coefficients and the observed p-values were checked. Comparing the accuracy of the derived models clarifies that the quadratic one is the most reliable approach for estimating both %E and %E_eff_^56, 69^. Equations (4) and (5) express the derived quadratic approaches for predicting the %E and %E_eff_, respectively.

4 $$\% {\text{E }} = { 14}.{95 }{-}{ 4}.{\text{15 Q }} + { 1}.{\text{38 C }} + { 4}.{\text{27 pH }}{-}{ 1}.{\text{55 Q }} \times {\text{ C }} + { 1}.{\text{1 Q }} \times {\text{ pH }} + { 2}.0{\text{1 Q}}^{{2}} {-}{ 4}.{\text{29 pH}}^{{2}} ,$$

5 $$\% {\text{E}}_{{{\text{eff}}}} = { 38}.{98 }{-}{ 13}.{\text{91 Q }} + { 3}.{5}0{\text{ C }} + { 5}.{\text{69 pH }}{-}{ 5}.{4}0{\text{ Q}} \times {\text{C }} + { 7}.{\text{61 Q}} \times {\text{pH }} + { 4}.0{\text{7 Q}}^{{2}} {-}{ 5}.{\text{76 pH}}^{{2}} .$$

It should be noted that those terms that possess a high p-value (p > > 0.05) were omitted from these final models. Indeed, the interaction terms of C^2^ and C × pH have been identified as unimportant factors and their associated terms have been removed from both %E and %E_eff_ models.

Table 3 introduces the numerical values of different factors required for appraising the models’ accuracy and evaluating features’ importance. The accuracy of developed models for estimating the %E and %E_eff_ can be checked using the numerical values of SSE (sum of squared errors) and MSE (mean squared errors) values. Furthermore, the p- and F-value indicate the importance of a distinct independent feature or the combination of different features for estimating a response (either %E or %E_eff_).

**Table 3:** The results of models’ accuracy and features importance checking.

Source	SSE		MSE		F-value		p-value	
	$\%E$	$\%{E}_{\mathrm{eff}}$	$\%E$	$\%{E}_{\mathrm{eff}}$	$\%E$	$\%{E}_{\mathrm{eff}}$	$\%E$	$\%{E}_{\mathrm{eff}}$
Model	403.95	2451.47	57.71	350.21	52.96	40.76	< 0.0001	< 0.0001
Q	137.53	1547.07	137.53	1547.07	126.22	180.06	< 0.0001	< 0.0001
C	15.18	97.72	15.18	97.72	13.93	11.37	0.0047	0.0082
pH	145.61	259.12	145.61	259.12	133.63	30.16	< 0.0001	0.0004
Q × C	9.64	116.53	9.64	116.53	8.85	13.56	0.0156	0.0051
Q × pH	4.88	231.34	4.88	231.34	4.48	26.93	0.0633	0.0006
Q^2^	17.11	69.85	17.11	69.85	15.71	8.13	0.0033	0.0191
pH^2^	77.77	140.22	77.77	140.22	71.37	16.32	< 0.0001	0.0029

Both the constructed models have a small p-value (p << 0.05) that the importance of their terms could be guaranteed. In addition, the F-value of %E and %E_eff_ models (i.e., 52.96 and 40.76) confirm that the models’ predictions are in complete agreement with the experimental records. The F-values also justify that the effect of all involved terms on responses is real. Moreover, the adjusted R^2^ of the %E and %E_eff_, models (i.e., 0.9763 and 0.9694) state that the modeling results have excellent compatibility with the experimental records.

#### pH impact on the extraction performance

**Figure 3:**
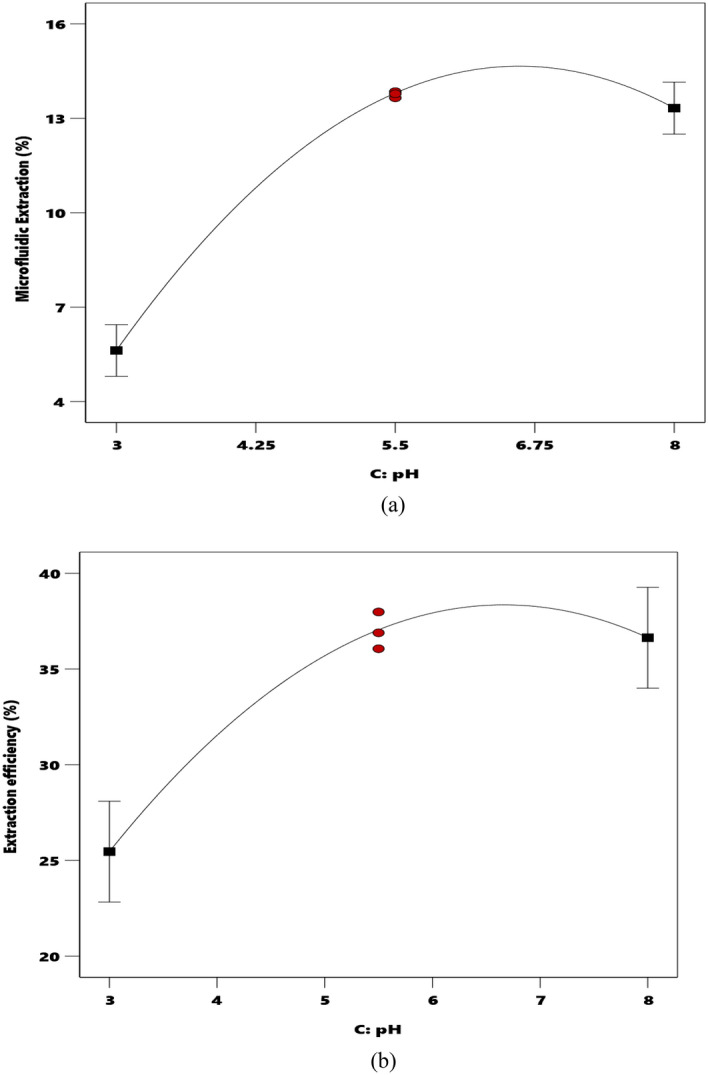
The dependency of the microfluidic extraction percentage (**a**) and extraction efficiency, (**b**) on initial pH (● design points ■ endpoints).

pH is one of the important parameters that influence the extraction performance of micro-size separation devices. Therefore, this section investigated the effect of the initial pH of the aquatic phase (3 < pH < 8) on the %E and %E_eff_. Figure 3a,b illustrate the pH effect on the %E and %E_eff_, respectively. It should be mentioned that this analysis is done using the constant values of 35 $\upmu \text{l}/\mathrm{min}$ and 0.01 M for the Q and C variables, respectively. The red dot is the design point and the square dot is the endpoint. The term “design points” refers to the non-center points in a block. The levels of the factors are coded so that the cube blocks contain design points with coordinate values all equal to ± 1, and center points at (0,0,0)^70^.

Figure 3a,b indicate that increasing the basic strength of the aquatic solution (the pH increase) decreases the slope of both %E-pH and %E_eff_-pH profiles. Accordingly, the distribution ratio decreases with increasing pH. Afterward, with increasing pH, the picrate anion affinity with the aquatic phase becomes higher than the organic phase. It can be concluded that the interface phenomenon reduces the amount of picrate anion. Since the reaction of extraction occurs in the interface, reducing the concentration of picrate anion results in decreasing the extraction rate. In summary, it can be said that the aquatic phase acidity has a high impact on the extraction performance of the studied microfluidic device.

#### Dependency of extraction performance on the DC18C6 dosage

**Figure 4:**
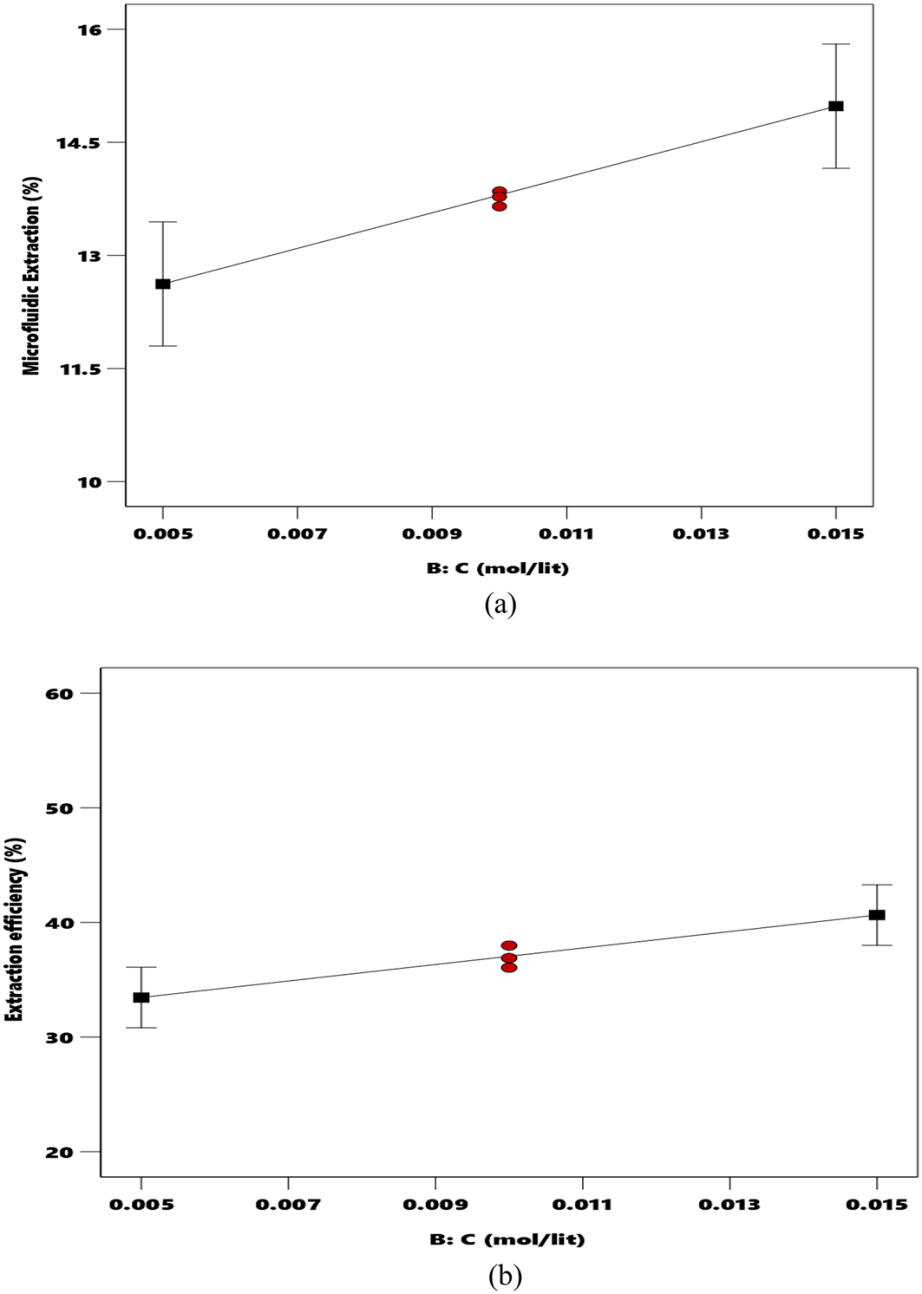
The effect of DC18C6 molarity on the microfluidic extraction percentage (**a**) and extraction efficiency (**b**) (● design points ■ endpoints).

The DC18C6 concentration is a significant variable for either experimental or modeling analyses of the microfluidic extraction percentage and extraction efficiency. In the experimental stage, the DC18C6 concentration was changed from 5 × 10^–3^ to 0.015 M. The effect of the DC18C6 concentration on the %E and %E_eff_ is depicted in Fig. 4a,b, respectively. These figures have been plotted using the pH 5.5 and aquatic phase flow rate = 35 $\mathrm{\mu l}/\mathrm{min}$ It can be seen that increasing the DC18C6 concentration almost linearly increases both %E_eff_ and %E. This could be related to DC18C6’s molecules that possibly form the complex with Ca^+2^. Equations (6) to (8) introduce the extraction/reaction mechanism of alkaline earth metals^71^:

6 $${{L}_{o}+M}^{+2}+2{A}^{-}\stackrel{1}{\iff }{MLA}_{2,o},$$

7 $${L}_{o}+{M}^{+2}+2{A}^{-}\stackrel{1}{\iff }{{A}_{o}^{-}+MLA}_{0}^{+},$$

8 $${L}_{o}+{M}^{+2}+2{A}^{-}\stackrel{1}{\iff }{ML}_{2}^{+2}+{2A}_{o}^{-2},$$

here picrate anion, DC18C6 (ligand), and the metal ion have been abbreviated with the ${A}^{-}$, $L$, and ${M}^{+2}$ symbols, respectively. The utilized subscript (i.e., o) denotes the organic phase. It can be concluded from the above reactions, the existing species interact with each other at the interface to form hydrophobic complexes.

The influence of DC18C6 concentration on %E is also experimentally studied from 5 × 10^–3^ to 0.015 and M (see Fig. 4a). This figure indicates that the DC18C6 concentration produces no notable change in the achieved values of %E in the proposed micro-size separation device.

#### The flow rate effect on the extraction performance

**Figure 5:**
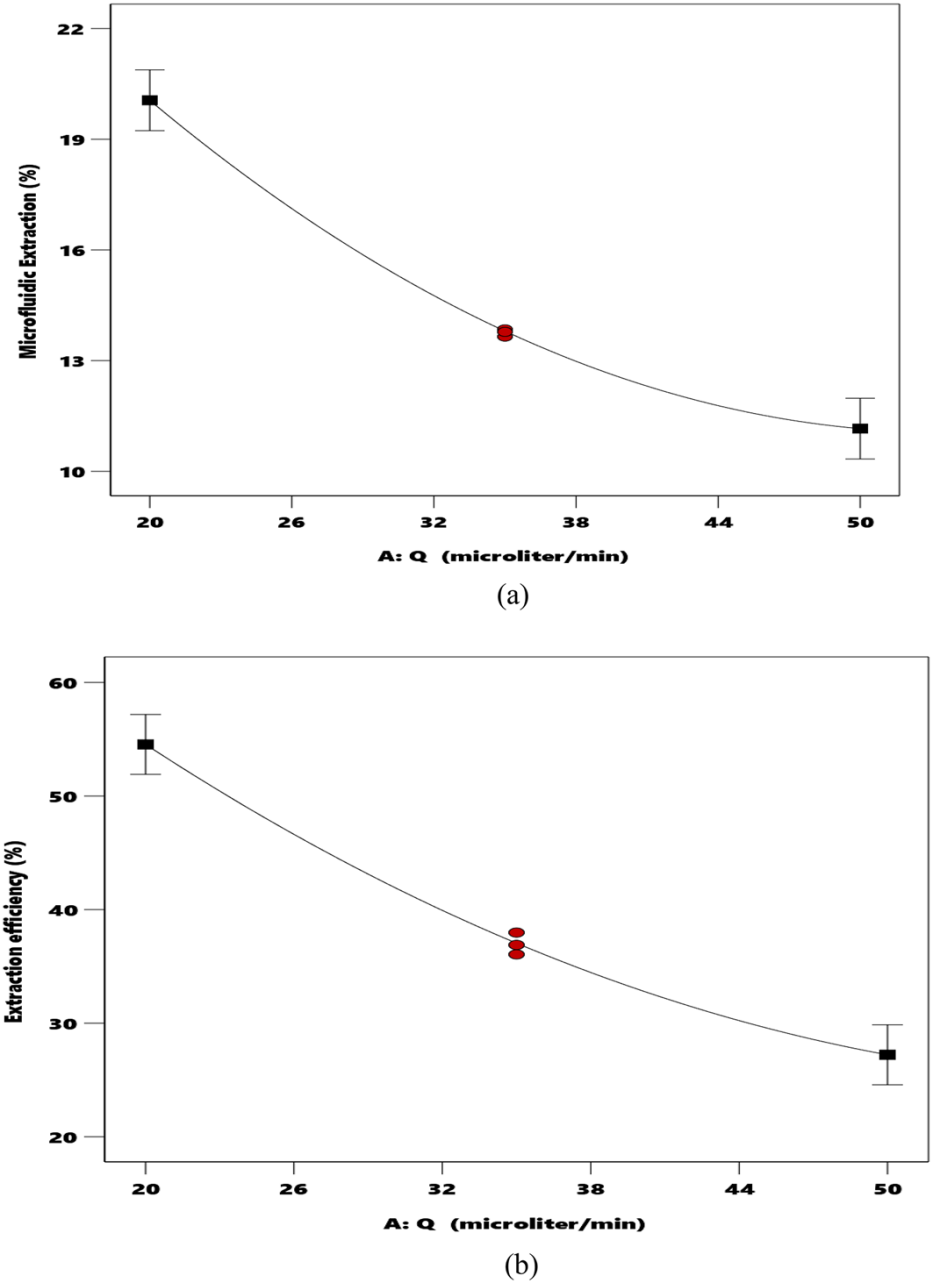
The variation of the microfluidic extraction percentage (**a**) and extraction efficiency (**b**) by aquatic phase flow rate (c and f) (● design points ■ endpoints).

Aquatic phase flow rate is another parameter that affects the achievable %E and %E_eff_ by the micro-size separation setup. Figure 5a,b display the profile of both %E and %E_eff_ versus the aquatic flow rate (at pH 5.5 and DC18C6 concentration = 0.01 M), respectively. These figures clearly indicate that increasing the flow rate gradually reduces the %E_eff_ and the %E. The small contact period between the involved phases and the low residence time of streams in the separation device is responsible for this observation.

#### The coupling effect of pH and flow rate on the extraction performance

**Figure 6:**
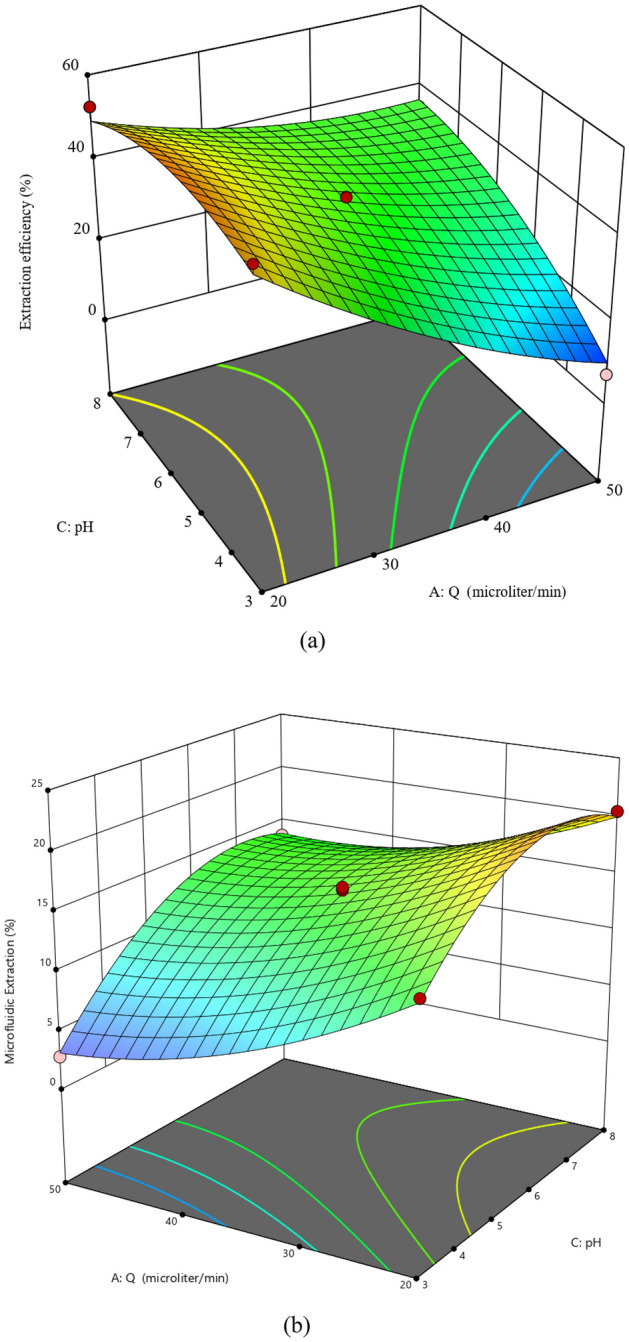
Three-dimensional graphs showing the couple effect pH and Q on (**a**) %E_eff_ and (**b**) %E (● design points lower than the predicted value, ● design points higher than predicted value).

Figure 6A and 6b illustrate the pairing effect of the pH and flow rate on the extraction efficiency and microfluidic extraction percentage (at C = 0.01 M), respectively. The previously reported p-value = 0.0006 (see Table 3) shows that the coupling term that combined pH and flow rate is an important factor for determining extraction efficiency. It can also be concluded from Fig. 6a, the pH affects the achievable %E_eff_ at the constant flow rate (Q). In addition, the pH effect on the observed %E_eff_ at high flow rates is stronger than its effect at the lower ones. Moreover, these two three-dimensional graphs show that the optimal value of %E_eff_ occurs at pH = 5, while the %E reaches its maximum value at pH 6.5.

#### The coupling effect of flow rate and DC18C6 dosage on the extraction performance

**Figure 7:**
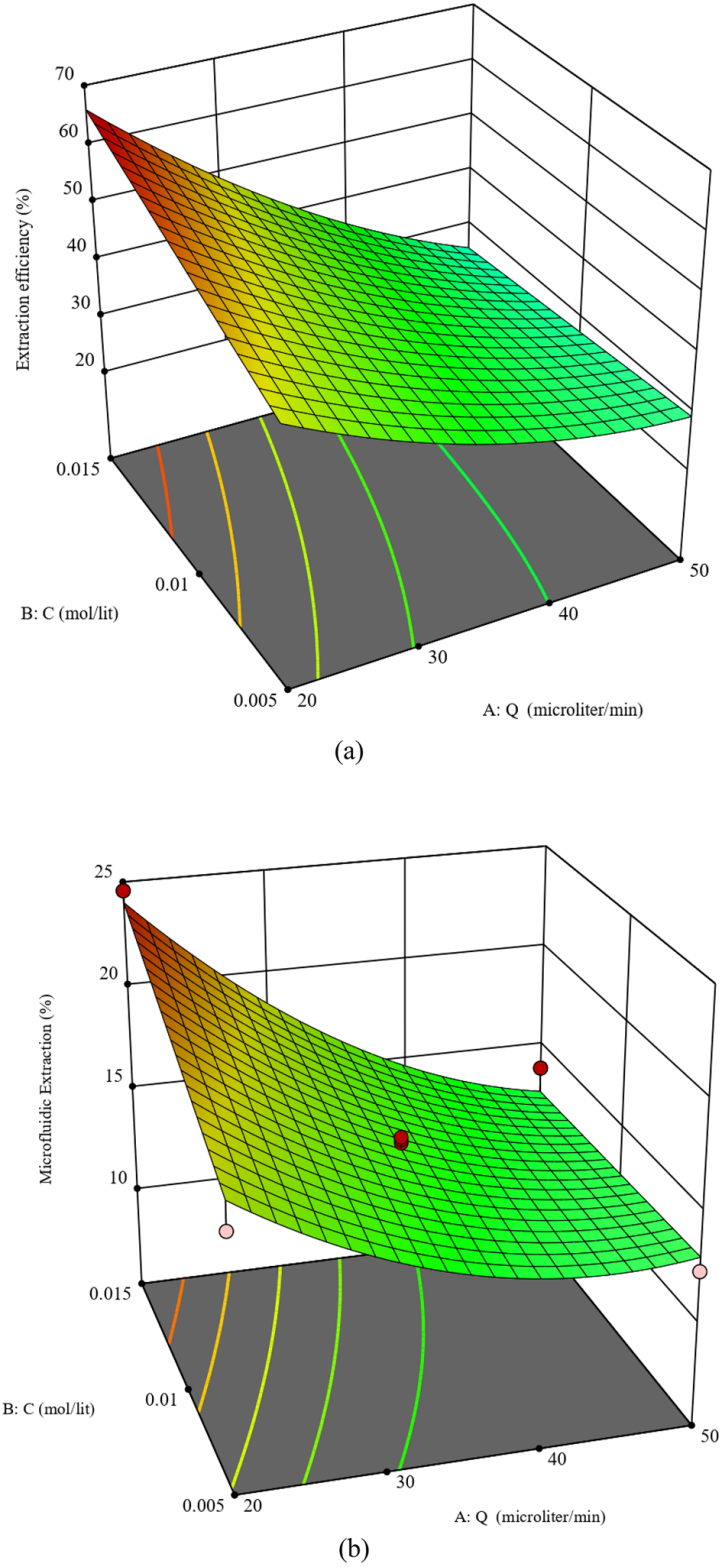
Three-dimensional graphs showing the coupling effect of C and Q on (**a**) %E_eff_ and (**b**) %E (● design points lower than the predicted value, ● design points higher than predicted value).

The influence of a term that combines DC18C6 concentration in the organic phase and aquatic flow rate on the separation performance (%E and the %E_eff_) of the proposed micro-scale setup is illustrated in Fig. 7a,b (pH 5.5). As it was also previously observed, both %E and %E_eff_ decrease when the aquatic phase flow rate increases. A short phase contact period as well as a relatively long diffusion length are responsible for the reducing trends of the extraction performance factors. These figures also state that the increasing rate of the microfluidic extraction percentage as well as extraction efficiency at high DC18C6 concentrations is faster than the observed rates at low DC18C6 concentrations. Moreover, increasing the extractor dosage (i.e., DC18C6) in the interface results in achieving better extraction performances via the micro-size setup. Although the maximum values of both %E and %E_eff_ appear at the maximum DC18C6 dosage, the concentration range of 0.01 to 0.015 M provides appropriate extraction when only the %E_eff_ is the desired response.

#### Determining optimal conditions

Solving the optimization problem associated with the micro-size setup operation helps find those conditions in which both %E and %E_eff_ have the highest possible values. Since these two extraction factors have an adverse-linear relationship with the aquatic phase flow rate (Fig. 5a,b), the smallest possible flow rates are the optimum condition. Therefore, the optimum value of 20 µl/min is suggested for the Q variable.

The numerical technique available in the utilized software helps optimize the other two independent features. The optimal values of 0.015 M and 6.34 are calculated for the DC18C6 dosage and pH of the aquatic phase, respectively. The extraction efficiency and microfluidic extraction at this optimized condition are 63.60% and 24.59%, respectively.

This section also applies the desirability function (DF) to simultaneously optimize the two involved response factors in our study. The numerical value of the DF can vary from 0 (completely unfavorable) to 1 (completely favorable). The achieved value of 0.991 for the DF approves that the optimum point has correctly been identified. A new experiment that is done at this optimum condition (also known as the confirmation test) provides 63.60 and 24.59 for the %E_eff_ and the %E, respectively.

#### Comparison with the literature

This section compares the extraction efficiency of the constructed micro-size device in the current study and those suggested by Abdullahi et al.^56^. Under the same operating conditions, the microfluidic extraction percentage as well as extraction efficiency of our setup is better than those reported in the literature^56^. Indeed, the achievable %E and %E_eff_ values by the serpentine microfluidic system are higher than those obtained by the Y-shape microfluidic system^56^.

## Conclusions

This research study proposes a systematic micro-size device for efficiently extracting calcium ions from the aquatic medium. Our suggested method can also be applied to extract other elements like alkaline and alkaline earth metals. The microfluidic extraction of 24.59% has been achieved by using this method at a residence time of 4.2 s. Moreover, liquid–liquid extraction with the traditional method needs at least 3 min for obtaining the same amount of extraction. Both modeling and optimization stages have been accomplished by applying the BBD method to the experimental records. Three independent variables, i.e., pH and flow rate of the aquatic phase and DC18C6 dosage in the organic phase are incorporated in the modeling and optimization of the %E and %E_eff_ response factors. The optimum value of pH and flow rate of the aquatic phase are 6.25 and 20 µl/min, respectively. Furthermore, the optimized value of DC18C6 dosage in the organic phase is 0.015 M. Based on the findings, the extraction efficiency = 63.60% has also been achieved for the Ca^2+^ separation at this optimum condition.

## Data Availability

The datasets used and/or analyzed during the current study are available from the corresponding author on reasonable request.

## References

[1] Jepson B, DeWitt R (1976). Separation of calcium isotopes with macrocyclic polyether calcium complexes. J. Inorg. Nucl. Chem..

[2] Fujii Y (2010). Mass dependence of calcium isotope fractionations in crown-ether resin chromatography. Isot. Environ. Health Stud..

[3] Karbasi E (2017). Experimental and numerical study of air-gap membrane distillation (AGMD): Novel AGMD module for oxygen-18 stable isotope enrichment. Chem. Eng. J..

[4] Manh TD, Bahramkhoo M, Barzegar Gerdroodbary M, Nam ND, Tlili I (2021). Investigation of nanomaterial flow through non-parallel plates. J. Therm. Anal. Calorim..

[5] Manh TD (2021). Computational simulation of variable magnetic force on heat characteristics of backward-facing step flow. J. Therm. Anal. Calorim..

[6] Amini Y, Ghazanfari V, Shadman MM, Heydari M, SayahAlborzi Z (2023). Optimization of liquid–liquid extraction of calcium with a serpentine microfluidic device. Int. Commun. Heat Mass Transf..

[7] Hazama, R. *et al.* Challenge on Ca-48 enrichment for candles double beta decay experiment. Preprint at http://arXiv.org/0710.3840 (2007).

[8] Umehara S (2015). A basic study on the production of enriched isotope 48Ca by using crown-ether resin. Progr. Theor. Exp. Phys..

[9] Moradi R, Karimi-Sabet J, Shariaty-Niassar M, Amini Y (2016). Air gap membrane distillation for enrichment of H218O isotopomers in natural water using poly (vinylidene fluoride) nanofibrous membrane. Chem. Eng. Process..

[10] Moradi R, Monfared SM, Amini Y, Dastbaz A (2016). Vacuum enhanced membrane distillation for trace contaminant removal of heavy metals from water by electrospun PVDF/TiO 2 hybrid membranes. Korean J. Chem. Eng..

[11] Moradi R, Karimi-Sabet J, Shariaty-niassar M, Amini Y (2016). Experimental investigation of nanofibrous poly (vinylidene fluoride) membranes for desalination through air gap membrane distillation process. Korean J. Chem. Eng..

[12] Kishimoto T, Matsuoka K, Fukumoto T, Umehara S (2015). Calcium isotope enrichment by means of multi-channel counter-current electrophoresis for the study of particle and nuclear physics. Progr. Theor. Exp. Phys..

[13] Gussone N, Dietzel M (2016). Calcium Stable Isotope Geochemistry.

[14] Ahmadi-Motlagh M, Amini Y, Karimi-Sabet J (2021). Experimental study of nitrogen isotope separation by ion-exchange chromatography: Effect of process factors. J. Radioanal. Nucl. Chem..

[15] Sheikholeslami M, Farshad SA, Gerdroodbary MB, Alavi AH (2022). Impact of new multiple twisted tapes on treatment of solar heat exchanger. Eur. Phys. J. Plus.

[16] Sheikholeslami M, Gerdroodbary MB, Shafee A, Tlili I (2020). Hybrid nanoparticles dispersion into water inside a porous wavy tank involving magnetic force. J. Therm. Anal. Calorim..

[17] Asadi Saghandi, H., Amini, Y. & Karimi-sabet, J. Hydrodynamic and mass transfer simulation of two immiscible phases in Y-Y and Spiral microchannels. *Nashrieh Shimi va Mohandesi Shimi Iran* (2021).

[18] Sattari-Najafabadi M, Esfahany MN (2021). A liquid–liquid microreactor for the intensification of hexavalent chromium removal from wastewaters. J. Environ. Chem. Eng..

[19] Sattari-Najafabadi M, Esfahany MN (2020). Hexavalent chromium extraction from aqueous solutions in a liquid–liquid slug flow microreactor. Chem. Eng. Process.-Process Intensif..

[20] Sattari-Najafabadi M, Esfahany MN, Wu Z, Sunden B (2018). Mass transfer between phases in microchannels: A review. Chem. Eng. Process.-Process Intensif..

[21] Barzegar Gerdroodbary M (2020). Application of neural network on heat transfer enhancement of magnetohydrodynamic nanofluid. Heat Transf. Asian Res..

[22] Templeton DM (2000). Guidelines for terms related to chemical speciation and fractionation of elements. Definitions, structural aspects, and methodological approaches (IUPAC Recommendations 2000). Pure Appl. Chem..

[23] Marsousi S, Karimi-Sabet J, Moosavian MA, Amini Y (2019). Liquid-liquid extraction of calcium using ionic liquids in spiral microfluidics. Chem. Eng. J..

[24] Sattari-Najafabadi M, Esfahany MNN (2017). Intensification of liquid–liquid mass transfer in a circular microchannel in the presence of sodium dodecyl sulfate. Chem. Eng. Process..

[25] Sattari-Najafabadi M, Nasr Esfahany M, Wu Z, Sundén B (2017). The effect of the size of square microchannels on hydrodynamics and mass transfer during liquid-liquid slug flow. AIChE J..

[26] Dastbaz A, Karimi-Sabet J, Ahadi H, Amini Y (2017). Preparation and characterization of novel modified PVDF-HFP/GO/ODS composite hollow fiber membrane for Caspian Sea water desalination. Desalination.

[27] Burns J, Ramshaw C (2001). The intensification of rapid reactions in multiphase systems using slug flow in capillaries. Lab Chip.

[28] Ciceri D, Perera JM, Stevens GW (2014). The use of microfluidic devices in solvent extraction. J. Chem. Technol. Biotechnol..

[29] Kashid MN, Gupta A, Renken A, Kiwi-Minsker L (2010). Numbering-up and mass transfer studies of liquid–liquid two-phase microstructured reactors. Chem. Eng. J..

[30] Lubej M (2015). Microfluidic droplet-based liquid–liquid extraction: Online model validation. Lab Chip.

[31] Kenig EY, Su Y, Lautenschleger A, Chasanis P, Grünewald M (2013). Micro-separation of fluid systems: A state-of-the-art review. Sep. Purif. Technol..

[32] Wang N, Zhao R, Zhang L, Guan X (2022). Molecular insights into the adsorption of chloride ions in calcium silicate hydrate gels: The synergistic effect of calcium to silicon ratio and sulfate ion. Microporous Mesoporous Mater..

[33] Li T (2022). Simultaneous removal of sulfate and nitrate from real high-salt flue gas wastewater concentrate via a waste heat crystallization route. J. Clean. Prod..

[34] Yin S (2015). Microfluidic solvent extraction of La (III) with 2-ethylhexyl phosphoric acid-2-ethylhexyl ester (P507) by a microreactor. Chem. Eng. Process..

[35] Sadeghi A, Amini Y, Saidi MH, Chakraborty S (2014). Numerical modeling of surface reaction kinetics in electrokinetically actuated microfluidic devices. Anal. Chim. Acta.

[36] Sadeghi A, Amini Y, Saidi MH, Yavari H (2015). Shear-rate-dependent rheology effects on mass transport and surface reactions in biomicrofluidic devices. AIChE J..

[37] Xu W (2022). Electrostatic atomization minimum quantity lubrication machining: From mechanism to application. Int. J. Extrem. Manuf..

[38] Liu W (2008). Treatment of CrVI-containing Mg (OH) 2 nanowaste. Angew. Chem..

[39] Kriel FH, Woollam S, Gordon RJ, Grant RA, Priest C (2016). Numbering-up Y-Y microfluidic chips for higher-throughput solvent extraction of platinum (IV) chloride. Microfluid. Nanofluid..

[40] Gao T (2021). Grindability of carbon fiber reinforced polymer using CNT biological lubricant. Sci. Rep..

[41] Gao T (2022). Fiber-reinforced composites in milling and grinding: Machining bottlenecks and advanced strategies. Front. Mech. Eng..

[42] Wang X (2022). Tribology of enhanced turning using biolubricants: A comparative assessment. Tribol. Int..

[43] Aota A, Mawatari K, Kitamori T (2009). Parallel multiphase microflows: Fundamental physics, stabilization methods and applications. Lab Chip.

[44] Asl YA, Yamini Y, Seidi S (2016). Development of a microfluidic-chip system for liquid–phase microextraction based on two immiscible organic solvents for the extraction and preconcentration of some hormonal drugs. Talanta.

[45] Berduque A, O’Brien J, Alderman J, Arrigan DWM (2008). Microfluidic chip for electrochemically-modulated liquid∣liquid extraction of ions. Electrochem. Commun..

[46] Castell OK, Allender CJ, Barrow DA (2009). Liquid–liquid phase separation: Characterisation of a novel device capable of separating particle carrying multiphase flows. Lab Chip.

[47] Zhao Y, Chen G, Yuan Q (2006). Liquid-liquid two-phase flow patterns in a rectangular microchannel. AIChE J..

[48] Squires TM, Quake SR (2005). Microfluidics: Fluid physics at the nanoliter scale. Rev. Mod. Phys..

[49] Yao X, Zhang Y, Du L, Liu J, Yao J (2015). Review of the applications of microreactors. Renew. Sustain. Energy Rev..

[50] Scheiff F, Mendorf M, Agar D, Reis N, Mackley M (2011). The separation of immiscible liquid slugs within plastic microchannels using a metallic hydrophilic sidestream. Lab Chip.

[51] Kreutzer MT, Kapteijn F, Moulijn JA, Kleijn CR, Heiszwolf JJ (2005). Inertial and interfacial effects on pressure drop of Taylor flow in capillaries. AIChE J..

[52] Hellé G, Mariet C, Cote G (2015). Liquid–liquid extraction of uranium (VI) with Aliquat® 336 from HCl media in microfluidic devices: Combination of micro-unit operations and online ICP-MS determination. Talanta.

[53] Logtenberg H, Lopez-Martinez MJ, Feringa BL, Browne WR, Verpoorte E (2011). Multiple flow profiles for two-phase flow in single microfluidic channels through site-selective channel coating. Lab Chip.

[54] Jovanović J (2012). Liquid–liquid flow in a capillary microreactor: Hydrodynamic flow patterns and extraction performance. Ind. Eng. Chem. Res..

[55] Di Miceli Raimondi N, Prat L, Gourdon C, Tasselli J (2014). Experiments of mass transfer with liquid–liquid slug flow in square microchannels. Chem. Eng. Sci..

[56] Abdollahi P, Karimi-Sabet J, Moosavian MA, Amini Y (2020). Microfluidic solvent extraction of calcium: Modeling and optimization of the process variables. Sep. Purif. Technol..

[57] Heydarzadeh M, Givianrad MH, Heydari R, Aberoomand Azar P (2019). Salt-assisted liquid–liquid extraction in microchannel. J. Sep. Sci..

[58] Tang J, Zhang X, Cai W, Wang F (2013). Liquid–liquid extraction based on droplet flow in a vertical microchannel. Exp. Thermal Fluid Sci..

[59] Singh K, Renjith A, Shenoy K (2015). Liquid–liquid extraction in microchannels and conventional stage-wise extractors: A comparative study. Chem. Eng. Process..

[60] Jahromi PF, Karimi-Sabet J, Amini Y (2018). Ion-pair extraction-reaction of calcium using Y-shaped microfluidic junctions: An optimized separation approach. Chem. Eng. J..

[61] Jahromi PF, Karimi-Sabet J, Amini Y, Fadaei H (2017). Pressure-driven liquid-liquid separation in Y-shaped microfluidic junctions. Chem. Eng. J..

[62] Ferreira SC (2007). Box–Behnken design: An alternative for the optimization of analytical methods. Anal. Chim. Acta.

[63] Cao Y, Kamrani E, Mirzaei S, Khandakar A, Vaferi B (2022). Electrical efficiency of the photovoltaic/thermal collectors cooled by nanofluids: Machine learning simulation and optimization by evolutionary algorithm. Energy Rep..

[64] Esmaeili-Faraj S, Hassanzadeh A, Shakeriankhoo F, Hosseini S, Vaferi B (2021). Diesel fuel desulfurization by alumina/polymer nanocomposite membrane: Experimental analysis and modeling by the response surface methodology. Chem. Eng. Process.-Process Intensif..

[65] Yang M (2019). Predictive model for minimum chip thickness and size effect in single diamond grain grinding of zirconia ceramics under different lubricating conditions. Ceram. Int..

[66] Bezerra MA, Santelli RE, Oliveira EP, Villar LS, Escaleira LA (2008). Response surface methodology (RSM) as a tool for optimization in analytical chemistry. Talanta.

[67] Yang M (2017). Maximum undeformed equivalent chip thickness for ductile-brittle transition of zirconia ceramics under different lubrication conditions. Int. J. Mach. Tools Manuf..

[68] Zhang J (2018). Experimental assessment of an environmentally friendly grinding process using nanofluid minimum quantity lubrication with cryogenic air. J. Clean. Prod..

[69] Hashemipour N (2018). Experimental and simulation investigation on separation of binary hydrocarbon mixture by thermogravitational column. J. Mol. Liq..

[70] Lenth RV (2010). Response-surface methods in R, using rsm. J. Stat. Softw..

[71] Kudo Y, Takeuchi T (2014). On the interfacial potential differences for the extraction of alkaline-earth metal picrates by 18-crown-6 ether derivatives into nitrobenzene. J. Thermodyn. Catal..

